# Advances in tumor-associated macrophage-mediated chemotherapeutic resistance in glioma

**DOI:** 10.3389/fcell.2025.1676338

**Published:** 2025-09-26

**Authors:** Xuebo Liu, Qi Yu

**Affiliations:** Department of Neurosurgery, Shengjing Hospital of China Medical University, Shenyang, China

**Keywords:** tumor-associated macrophages, glioma, drug resistance, reprogramming, tumor microenvironment

## Abstract

Tumor-associated macrophages (TAMs) are a dominant immune component within the glioma microenvironment and are increasingly recognized as key contributors to therapeutic resistance, the major challenge in glioma management. Understanding their role is critical for developing novel therapies. This review synthesizes current knowledge on TAM-mediated chemoresistance in glioma. TAMs originate from bone marrow-derived monocytes and resident microglia, exhibiting significant heterogeneity and plasticity, particularly between pro-inflammatory (M1) and pro-tumorigenic (M2) phenotypes. M2-like TAMs drive resistance through multiple mechanisms: (1) Modulating drug metabolism/clearance (e.g., via CYP450 enzymes and P-glycoprotein); (2) Secreting protumor factors (TNF-α, ILs like IL-4/IL-6/IL-10, chemokines like CCL5/CCL22, growth factors like VEGF/EGF) that activate survival pathways, induce immunosuppression, promote angiogenesis, and enhance epithelial-mesenchymal transition (EMT); (3) Interacting with glioma stem cells (GSCs) to maintain stemness; (4) Facilitating microenvironmental adaptation (e.g., hypoxia/HIF-1α response); (5) Remodeling the extracellular matrix (ECM) via MMPs, increasing stiffness and impairing drug penetration. Targeting TAMs offers promising approaches to overcome resistance. Strategies include: (1) Reprogramming M2 to M1 phenotypes using agonists (TLR, STING, CD40) or inhibitors (STAT3/STAT6); (2) Metabolic modulation (targeting glycolysis, fatty acid oxidation, glutaminolysis); (3) Blocking recruitment axes (CCL2/CCR2, CSF-1/CSF-1R, CXCL12/CXCR4); (4) Depleting M2-TAMs (e.g., trabectedin, CAR-T cells, M2pep-drugs); (5) Enhancing phagocytosis (anti-SIRPα/CD47, anti-SIGLEC). TAMs are pivotal mediators of chemoresistance in glioma through diverse molecular and cellular mechanisms. Targeting TAM recruitment, polarization, function, or metabolism represents a promising therapeutic avenue. However, the complexity of the glioma microenvironment and blood-brain barrier necessitate combination strategies for clinical translation. Further research is needed to optimize specificity and overcome challenges like compensatory pathways and drug delivery.

## 1 Introduction

Glioblastoma (GBM) is a highly aggressive, WHO Grade IV astrocytic tumor and the most prevalent malignant primary brain cancer in adults, characterized by extensive invasiveness and a profoundly poor prognosis. Malignant tumors known as gliomas are developed in the central nervous system and from glial cells, including astrocytes, oligodendrocytes, and ventricular cells, in the brain or spinal cord (Jiang et al., 2021; Figarella-Branger et al., 2022). In the treatment of gliomas, the standard therapeutic strategy consists mainly of surgical resection, radiotherapy, and chemotherapy (Rong et al., 2022). The combination of these treatments is designed to minimize the tumor burden and prolong patient survival. However, the complexity and heterogeneity of gliomas usually results in poor patient outcomes, and gliomas have an extremely high recurrence rate, with many patients experiencing tumor recurrence shortly after initial treatment (Ya et al., 2022). The refractory and recurrent nature of gliomas involves numerous complex mechanisms in which the tumor microenvironment (TME) predominates (Yang et al., 2020). The TME includes tumor cells, a variety of nontumor cells, the extracellular matrix (ECM), and a variety of biomolecules (Bied et al., 2023). Glioblastoma-associated macrophages/microglia (GAMs) constitute the most abundant immune population in the TME, accounting for 50% of the total number of cells in the GBM (Xuan et al., 2021; Simonds et al., 2021). The abundance of TAMs is usually correlated with prognosis; TAM abundance is positively correlated with glioma grade and negatively correlated with survival (Komohara et al., 2008; Urbantat et al., 2021a). Macrophages are a double-edged sword in the development of gliomas. Macrophages have the ability to kill tumor cells and activate tumor resistance mechanisms (Larionova et al., 2019). Tumor-associated macrophages (TAMs) contribute synergistically to chemotherapy resistance and disease progression in gliomas through diverse mechanisms. TAMs express high levels of drug-metabolizing enzymes (e.g., CYP450 family members) and efflux transporters such as P-glycoprotein, facilitating the metabolic clearance of chemotherapeutic drugs and reducing their intratumoral concentrations (Liu et al., 2024a; Guo Y. et al., 2024; Tran et al., 2024). Furthermore, TAMs promote an immunosuppressive microenvironment via the adenosine/A2AR/mTORC signaling axis, which depletes tryptophan and impairs T-cell function, as well as through glucose/HBP pathway-driven secretion of cathepsin B (Ding et al., 2025; Shi et al., 2022). TAMs also secrete various cytokines—including TNF-α, IL-4, IL-6, IL-10, CCL5, CCL22, and VEGF—that sustain activation of pro-survival pathways such as NF-κB, JAK/STAT, and PI3K/Akt in tumor cells, thereby inhibiting apoptosis and enhancing DNA repair (Wang et al., 2024a). Additionally, TAM-derived exosomes carry non-coding RNAs (e.g., miR-21, miR-27a-3p, and lncRNA SBF2-AS1) that are internalized by tumor cells, reinforcing their drug-resistant phenotype (Hu et al., 2025). Through the secretion of matrix metalloproteinases (MMPs) and cathepsin B, TAMs remodel the extracellular matrix (ECM), increasing tissue stiffness and limiting drug penetration (Simoes et al., 2020). Hypoxia further amplifies this process by activating HIF-1α signaling, which upregulates VEGF expression and promotes angiogenesis and metabolic adaptation (Chen et al., 2019). TAMs further diminish chemotherapy efficacy by establishing a highly immunosuppressive microenvironment through BRD4/PAI-1-mediated apoptosis inhibition and DNA repair, TGF-β/IL-10-driven immunosuppressive microenvironment formation, and epigenetic regulation (BRD4-driven PAI-1 expression) (Wang et al., 2024a; Pan et al., 2025). Thus, blocking the protumor activity of macrophages is an important component of future immunotherapy. In this review, we discuss how TAMs, important components of the TME, promote tumor progression and may therefore be useful as therapeutic targets.

## 2 Origin of TAMs

**FIGURE 1:**
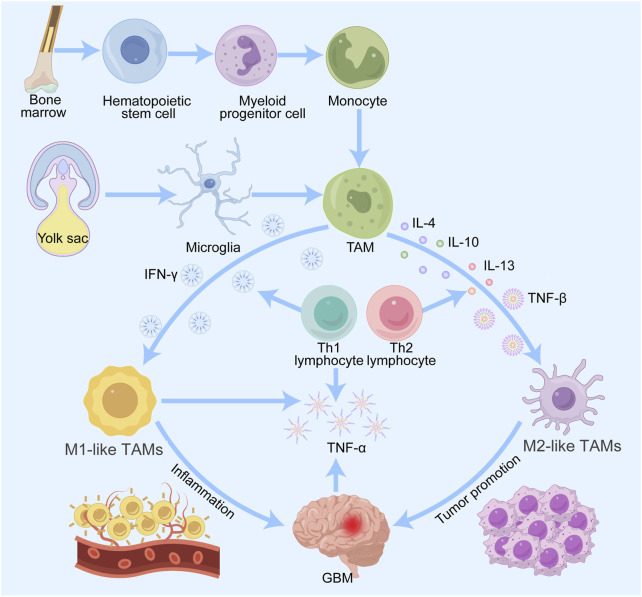
Origin and differentiation of TAMs in gliomas. Macrophages are derived from monocytes of bone marrow hematopoietic stem cells, and microglia are derived from progenitor cells of the embryonic yolk sac. Both types of cells enter the CNS and are recruited by glioma cells to become TAMs. TAMs are induced by various factors to differentiate into M1 and M2 types. M1 macrophages promote inflammation and M2 macrophages promote tumor progression, metastasis and angiogenesis.

TAMs in gliomas are predominantly derived from microglia and macrophages in the yolk sac and bone marrow (Xu et al., 2022). TAMs are predominantly derived from the bone marrow, and the precursor cells of these macrophages are monocytes, which migrate through the bloodstream into the tumor tissue and thereafter change into macrophages in the local environment (Li D. et al., 2024). Monocyte production and maturation in the bone marrow are regulated by a variety of cytokines and growth factors, such as granulocyte‒macrophage colony‒stimulating factor (GM-CSF) and interleukin-6 (IL-6) (Li et al., 2021). Once in the TME, these monocytes are attracted to chemokines secreted by the tumor cells and thus colonize and transform into TAMs (Figure 1). In addition to bone marrow-derived monocytes, TAMs also originate from embryonic precursor cells, which are usually derived from erythromedullary progenitors in the yolk sac during early embryonic development and migrate postnatally to various tissues, including tumor tissues (Andersen et al., 2022). These embryonic-derived macrophages are known as tissue-resident macrophages. In addition, factors related to the metabolic state of the tumor cells, the hypoxic environment and the remodeling of the extracellular matrix, also affect macrophage origin and polarization (Khan F. et al., 2023). For example, under hypoxic conditions, macrophages may polarize toward the tumor-promoting M2 phenotype, further promoting tumor growth and metastasis (Yang et al., 2020). The sources of TAMs are complex and diverse, and the characteristics of the TME and the secretion status of cytokines play key roles in the formation and function of TAMs. Elucidating the origin and mechanism of action of TAMs is crucial for the advancement of tumor treatment strategies focusing on TAMs.

## 3 The role and characteristics of TAMs

### 3.1 Heterogeneity

As key components of the TME, TAMs are closely associated with inflammation and tumor development (Chim et al., 2023). TAMs show significant heterogeneity in the TME, based primarily on their origin, polarization, and function. The TAMs heterogeneity plays a crucial role in the emergence of drug resistance. These macrophages originate from diverse sources, including bone marrow-derived monocytes and tissue-resident macrophages, each of which exhibit distinct characteristics and functions in unique tissue environments (Laviron et al., 2022). Macrophages in the microenvironments of different types of tumors (e.g., breast cancer, colorectal cancer, and osteosarcoma) exhibit different molecular features and functions (Larionova et al., 2019).

#### 3.1.1 Functional polarization: M1 and M2 phenotypes

Cytokines, chemokines, and other immune cell interactions in the TME affect macrophage polarization status and function (Nasir et al., 2023). Mills and colleagues firstly categorize macrophages into M1 and M2 type. M1 macrophages, which are usually considered pro-inflammatory macrophages, are activated by pro-inflammatory factors such as interferon gamma (IFN-γ) and bacterial endotoxin. Activated M1 macrophages can effectively phagocytose pathogens and tumor cells and promote antitumor immunity by generating reactive oxygen species (ROS) and nitric oxide (NO) and by secreting pro-inflammatory mediators, such as IL-1, IL-12, and IL-18 (Guo et al., 2022). In contrast, M2 macrophages, often referred to as inflammation-suppressive macrophages, can be activated by cytokines such as IL-4 or IL-13 and promote inflammation abatement and pathogen immune escape through the secretion of anti-inflammatory cytokines such as IL-10 and TGF-β, which facilitate tissue regeneration, suppress antitumor immune responses, promote angiogenesis and cell migration, and aid in tumor growth and metastasis (Boutilier and Elsawa, 2021; Sun et al., 2024). Therefore, in tumor therapy, M1-like macrophages are usually considered beneficial because of their antitumor activity, whereas M2-like TAMs are considered targets for inhibition or reprogramming because of their role in promoting tumor progression.

#### 3.1.2 Heterogeneity and therapeutic resistance

The heterogeneity of TAMs manifests not only in their phenotypic diversity but also influences tumor cell states and treatment responses. Malignant glioma cells have been found to have four states: neural progenitor cell-like (NPC-like), oligodendrocyte progenitor cell-like (OPC-like), astrocyte-like (AC-like), and mesenchymal-like (MES-like) (Chen et al., 2023). A higher proportion of macrophages in MES-like tumor cells and concomitant treatment with macrophage-depleting chlorophosphates resulted in a significant reduction in MES-like glioma cells, suggesting a macrophage-induced transition to a MES-like state (Chu et al., 2024). By modulating the polarization state of macrophages, new immunotherapeutic approaches for glioma can be explored.

### 3.2 Plasticity

The plasticity of TAMs is mainly based on their polarization properties, as demonstrated by their ability to adjust their phenotype and in response to signaling the TME. Early studies suggested that TAMs can be classified into two polarization states: classically activated M1-like macrophages and alternatively activated M2-like TAMs (Yang et al., 2020). Single-cell sequencing and other technologies have revealed that the gene expression of M1- and M2-like TAMs is not entirely mutually exclusive; instead, there exists some degree of overlap and continuity (Boutilier and Elsawa, 2021). For example, TAMs in gliomas can express both M1- and M2-like marker genes, suggesting that they may possess both pro-inflammatory and anti-inflammatory properties, an expression pattern that is inconsistent with the traditional M1/M2 dichotomy (Zhu et al., 2023). Thus, TAMs are involved in a dynamic process of interconversion between the M1 and M2 types. This conversion is shaped by the cytokine environment, metabolites, pathogens, damage-associated molecular patterns (DAMPs), and other cell-to-cell interactions. Several subpopulations of M2 cells have been categorized as M2a, M2b, M2c, and M2d since the stimuli and transcriptional alterations occurring within the TME (Chamseddi et al., 2022). Accurate identification of TAM subtypes and their functions can contribute to the precise treatment of tumors (Table 1). The regulatory mechanisms governing the dynamic transition between M1 and M2 phenotypes in TAMs demonstrate both shared features and context-dependent variations across different cancers, with notable distinctions observed in gliomas compared to other tumor types. These differences arise primarily from unique TME compositions and tissue-specific characteristics. A hallmark of the glioma TME is its pervasive hypoxia, which not only stimulates tumor cells to secrete factors such as lactate and SPARC but also strongly polarizes TAMs toward an M2-like or hypoxia-associated macrophage (Hypo-TAM) phenotype (Liu et al., 2022; Wang et al., 2024b). This distinct TAM subpopulation highly expresses genes including ADM, thereby facilitating aberrant angiogenesis and enhancing immunosuppression (Wang et al., 2024b). Furthermore, TAMs in GBM display remarkable cellular heterogeneity and diverse origins: they consist of both brain-resident microglia and bone marrow-derived macrophages (BMDMs), which differ significantly in their transcriptomic profiles, functional properties, and signaling responses (Zhao et al., 2025). Single-cell RNA sequencing has identified multiple functionally distinct TAM subsets (e.g., Mo-TAM_inf and Mg-TAM_hom) and revealed their spatially organized distribution within specific anatomical niches—such as perivascular areas, hypoxic and necrotic regions, and invasive fronts—reflecting a complexity that surpasses that of most solid tumors (Wang et al., 2024b; Zhao et al., 2025). The presence of the blood-brain barrier (BBB) further shapes this relatively segregated immune milieu, not only limiting drug penetration but also fostering unique immune cell crosstalk and signaling pathways, such as TAM recruitment via the CX3CL1–CCR2 axis (Zhao et al., 2025; Franson et al., 2022). In contrast, TAM polarization in other malignancies—such as lung, breast, and colorectal cancers—is more commonly driven by canonical cytokine signaling pathways involving IL-4, IL-13, IL-10, and TGF-β (Xu et al., 2025). Although exosome-mediated regulation represents a common mechanism across various tumors (e.g., miR-1246 promoting M2 polarization in glioma), the molecular cargo and functional impact of exosomes are highly tumor-type specific (Qian et al., 2020; Jin and Bai, 2025; Wang et al., 2024c). At the level of metabolic reprogramming, lactate—a universal glycolysis byproduct—promotes M2 polarization broadly; however, the synergistic action of SPARC and lactate observed in gliomas exhibits relative specificity (Wang et al., 2024b). In other tumor types, alternative metabolic pathways such as arginine metabolism and fatty acid oxidation (FAO) may play more dominant roles in steering TAM polarization (Yang et al., 2025; Wang et al., 2024d). These differences profoundly influence the development of treatment strategies: glioma therapy requires greater focus on overcoming the blood-brain barrier, targeting hypoxia-associated specific subpopulations (such as Hypoxia-TAM), and combining with existing radiotherapy and chemotherapy regimens; while other solid tumors may more broadly utilize inhibitors targeting classical chemotaxis/polarization pathways (e.g., CCR2, CSF-1R, CD40) and combine them with immunotherapy approaches like immune checkpoint inhibitors.

**TABLE 1:** Comparison of markers and biological functions in different macrophage subtypes.

Macrophage subtype	Stimulator factors	Markers	Secreted molecules	Related signaling pathways	Functions
M1	IFN-γ, LPS, TNF-α, GM-CSF	CD40, CD64, CD68, CD80, CD86, MHCII, TLR2/4	iNOS, ROS, TNF-α, IL-1β, IL-6, IL-12, IL-23, CXCL9, CXCL10, CXCL11	JAK-STAT1 pathway, NF-κB pathway, MAPK pathway, HIF-1α pathway	Pro-inflammatory, tumor resistance, Immune stimulation
M2a	IL-4, IL-13	CD163, CD206, CD209, CCL17, CCL18, CCL22, Arg1, Dectin-1, FIZZ1	IL-4, IL-10, TGF-β, CCL1, CCL17, CCL18, CCL22, CCL24	STAT6 pathway,PI3K/Akt pathway,PPAR-γ pathway	Inhibits inflammation, promotes tissue repair, fibrosis, angiogenesis, parasite clearance, and immunosuppression
M2b	LPS + ICs	CD86, CCL1, SPHK1, TNF-α, IL-10, MHCII	IL-1, IL-6, IL-10, SPHK1, CCL1, TNF-α	STAT3 pathway,PI3Kγ/mTOR pathway	Immunoregulation, tumor progression
M2c	IL-10, TGF-β, glucocorticoids	CD163, TLR1, TLR8, MerTK, TGF-β, IL-10	IL-10, TGF-β, CCL16, CCL18, CXCL13	STAT3 pathway,TGF-β/Smad pathway	Immunosuppression, angiogenesis, efferocytosis
M2d	IL-6,TLR,LPS	CD163, VEGF, IL-10	IL-10, IL-6, CCL18, M-CSF, TGF-β	STAT3 pathway,PI3K/Akt pathway	Promotes tumor angiogenesis, invasion and metastasis, immune suppression, and tissue remodeling

### 3.3 TAMs in tumor development

**FIGURE 2:**
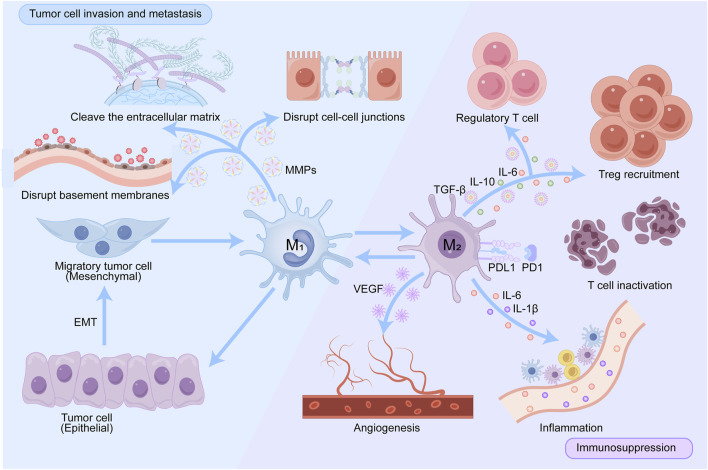
Pathways by which TAMs promote tumor progression. TAMs create a pro-tumor microenvironment in multiple ways: by producing various cytokines that promote regulatory T cells recruitment; by expressing PD-L1, blocking PD-1-induced T cell engagement and creating an inflammatory environment. VEGF secreted by activated TAMs promotes angiogenesis, thereby promoting tumor progression and metastasis. TAMs shape the extracellular matrix by producing proteases such as MMPs that degrade collagen fibers and disrupt connections between cells and between the matrixes.

Although TAMs have pro-tumor functions in tumor progression, they can also exert anti-tumor effects in glioma due to the complex heterogeneity and plasticity of tumors (Khan F. et al., 2023). This article explores the role of TAMs in promoting tumor occurrence and development from the following three aspects (Figure 2).

#### 3.3.1 Immunosuppression

As key immune regulatory cells in the TME, TAMs can suppress the immune system through a multiple of pathways. TAMs secrete immunosuppressive cytokines, including IL-10 and TGF-β, that impede T cell activation and proliferation while promoting the development of regulatory T cells (Tregs), thus attenuating antitumor immune responses (Monteleone et al., 2023). Furthermore, TAMs are able to express immune checkpoint molecules such as PD-L1 and PD-L2, which interact with PD-1 on T cells, leading to reduced T-cell activity, T-cell exhaustion and immune evasion (Zhang et al., 2023). In the TME, no cell exists in isolation. TAMs can interact directly or indirectly with dendritic cells, T cells, and B cells, causing them to change their functions and become supportive rather than antagonistic to tumor growth (Zhang et al., 2023; Sadhukhan and Seiwert, 2023). TAMs not only affect tumor growth and metastasis but also inhibit the body’s immune response through a variety of mechanisms and reduce the efficacy of immunotherapy. A comprehensive understanding of the functions and mechanisms of TAMs is crucial for the development of innovative tumor treatment strategies.

#### 3.3.2 Promotion of angiogenesis

Angiogenesis is a critical process in tumor progression as it is responsible for providing nutrients and oxygen required by tumors (Lopes-Coelho et al., 2021). Vascular endothelial growth factor (VEGF), angiopoietin, the Notch receptor, fibroblast growth factor (FGF), and epidermal growth factor (EGF) play an important role in the VEGF pathway (Vimalraj, 2022). TAMs promote endothelial cell migration and proliferation by secreting a variety of proangiogenic factors, such as VEGF, FGF2, and matrix metalloproteinases (MMPs), thereby stimulating neointima formation (Zhao et al., 2022). In addition, hypoxia areas often appear in tumor tissues, and upregulation of the hypoxia-dependent transcription factor HIF1α can promote VEGF expression, thereby promoting the proangiogenic function of TAMs (Chen et al., 2019). Currently, antivascular therapeutic drugs have been used in the treatment of glioma and effectively improve patients’ quality of life, but drug resistance remains an urgent problem.

#### 3.3.3 Promotion of tumor cell invasion and metastasis

TAMs reshape the TME and promote the growth and metastasis of tumor cells by releasing a variety of cytokines, chemokines, and enzymes. For example, TAMs can secrete MMPs, which can lyse the extracellular matrix and disrupt cell-cell junctions and basement membranes, ultimately helping tumor cells break through these barriers and achieve invasion (Bied et al., 2023; Yuan et al., 2023). In addition, M2-like TAMs enable tumor cells to gain greater invasive ability through epithelial‒mesenchymal transition (EMT) (Li et al., 2022). M2-like TAMs secrete EGF, a proinvasive factor that activates Smad2/3 and EMT-related transcription factors through the ERK1/2 and PI3K/Akt signaling pathways, thereby promoting the expression of EMT markers, such as increasing in N-cadherin and vimentin and decreasing of E-cadherin, changing characteristic of EMT (Kim et al., 2016; Karimi Roshan et al., 2019). EMT transforms tumor cells from an epithelial state to a mesenchymal state with greater migratory ability and promotes tumor cell invasion. EGF induces EMT through multiple signaling pathways, including the ERK1/2, Akt, and TGF-β signaling pathways. EGF-induced EMT plays an important role in tumor biology, especially in cancer metastasis and drug resistance. Interfering in these signaling pathways is expected to enhance the effectiveness of cancer treatments and reduce the reduction in tumor metastasis.

## 4 Multiple mechanisms of therapeutic resistance driven by M2-Like TAMs

### 4.1 Drug metabolism and clearance

TAMs significantly affect tumor therapeutic drugs by secreting drug-metabolizing enzymes and regulating drug transport. Research shows that TAMs in the TME can induce drug resistance through the following mechanisms: (1) TAMs are able to express a variety of drug-metabolizing enzymes, including cytochrome P450 enzymes (CYP450s) and other enzymes related to drug metabolism. CYP450s are mainly responsible for the metabolism of drugs and exogenous compounds and catalyze oxidative reactions, particularly the conversion of fat-soluble substances to facilitate excretion (Tran et al., 2024). TAMs secrete cytokines such as IL-10 and TGF-β, which can increase the activity of the CYP450 system in tumor cells, thereby accelerating the metabolism of tumor therapeutic drugs and reducing their effective concentrations. This increase in metabolism may lead to resistance to chemotherapeutic drugs, thereby affecting the effectiveness of treatment (Liu et al., 2024a). (2) TAMs can internalize and excrete drugs via transporter proteins, a function that can directly affect the distribution of drugs within tumor tissues. P-glycoprotein (P-gp) is an ATP-binding cassette (ABC) transporter protein encoded by multidrug resistance gene 1 (MDR1) (Guo Y. et al., 2024). The release of P-gp results in the export of chemotherapeutic from tumor cells, leading to chemoresistance, a process that is driven by the provision of energy through ATP hydrolysis; thus, P-gp activity is closely related to the intracellular ATP level (Guo Y. et al., 2024; Patel et al., 2024). Therefore, moderate induction of mitochondrial dysfunction or inhibition of glutamine metabolism can be considered to decrease P-gp activity, thereby increasing chemotherapy sensitivity (Guo Y. et al., 2024; Lee et al., 2021; Yoo et al., 2020). P-gp also functions as a transporter protein in the blood‒brain barrier, which can limit the entry of hazardous substances and certain drugs and protect the central nervous system from external influences (Chai et al., 2022). The expression and function of P-gp are regulated by multiple signaling pathways; e.g., in ovarian cancer, miR-1246 activates P-gp by targeting the Cav1/P-gP/PRPS2/M2-like macrophage signaling pathway to inhibit the uptake and transport of paclitaxel (Ghafouri-Fard et al., 2021). P-gp significantly contributes to multidrug resistance in tumors, primarily through its overexpression, which enables cancer cells to evade the effects of chemotherapy drugs.

In addition to mediating drug resistance through metabolic enzymes and transporter proteins, TAMs further promote chemoresistance by remodeling the tumor microenvironment via the secretion of diverse soluble factors—including cytokines, chemokines, and growth factors. These molecules activate key pro-survival, proliferative, and immunomodulatory signaling pathways in cancer cells, ultimately leading to enhanced treatment evasion. The following sections detail how these factors contribute to therapy resistance.

### 4.2 Tumor necrosis factor-α (TNF-α)

TNF-α is an important cytokine, mainly produced by macrophages and is widely involved in the inflammatory response and immune regulation. TNF-α interacts with two major receptors, TNF receptor 1 (TNFR1) and TNF receptor 2 (TNFR2), each of which has distinct biological roles in various cell types. TNFR1 mainly mediates apoptosis, the inflammatory response, and the immune response, and its signaling occurs mainly through the activation of transcription factors such as NF-κB and AP-1, which trigger the upregulation of genes associated with the inflammatory response (Jang et al., 2021). TNFR2 is usually associated with cell proliferation and survival and is expressed mainly in immune cells, which are able to promote the function and survival of regulatory T cells (Moatti and Cohen, 2021). Studies have shown that the presence of large amounts of TNF-α in the TME may lead to tumor resistance (Cruceriu et al., 2020). M2-like TAMs TNF-α and other protumorigenic cytokines that activate indoleamine 2,3-dioxygenase 1 (IDO1), which can increase the resistance of tumor cells to bevacizumab (Liu Y. et al., 2020). M2-like TAMs can trigger the NF-kβ pathway via the TME, leading to significant increase in CXCL1 and CXCL2 levels, which are crucial for promoting metastasis and chemoresistance (Urbantat et al., 2021b).

### 4.3 Interleukins (ILs)

Interleukins are a class of cytokines secreted by immune cells that are involved in the regulation of the immune response. Different ILs play different roles in the TME; some promote tumor growth, whereas others may inhibit tumor progression. For example, IL-1, IL-4, and IL-6 play a tumor-promoting role in tumor progression. IL-1 has been shown to promote tumor growth and metastasis; this result was confirmed in transplantation studies of mouse and human tumors. The tumor-promoting effects of IL-1α and IL-1β involve complex mechanisms, including immunosuppression and tumor-promoting myeloid cell recruitment (Garlanda and Mantovani, 2021). EGFR T790M-cis-L792F triggers the JAK/STAT3 signaling pathway and promotes the specific attachment of p-STAT3 to the IL-4 promoter, thereby increasing IL-4 production and release, facilitating M2-like macrophage polarization (Sun Y. et al., 2022). The binding of IL-6 to its receptor activates a variety of tumor-promoting pathways, including the JAK/STAT3, PI3K/AKT, and Ras/MAPK pathways. The activation of these pathways is closely related to cell proliferation, survival, and migration (Masjedi et al., 2018).

### 4.4 Chemokines

There are four major subfamilies of chemokines: CXC, CC, CX3C, and XC, which interact with G protein-coupled transmembrane receptors to exert their biological effects (Braoudaki et al., 2022). CCL5 from TAMs can play an immunosuppressive role by promoting PD-L1 expression through the p65/STAT3 pathway (Liu C. et al., 2020). In addition, CCL5 can significantly promote EMT and the migration and invasion of prostate cancer cells (Huang et al., 2020). CCL22 recruits Tregs to tumor tissues via CCR4 and inhibits the function of effector T cells, leading to the escape of tumor cells from immune surveillance (Lecoq et al., 2022; Anz et al., 2015).

### 4.5 Growth factors

Growth factors are signaling molecules, usually in the form of peptides, that promote cell proliferation and differentiation. They activate a series of downstream signaling pathways by binding to receptors on the cell surface, thereby promoting cell growth and survival. Many cancer cells can synthesize and secrete their own growth factors, a mechanism that allows cancer cells to promote their own growth and survival independently of the influence of external growth factors (Liu L. et al., 2025). VEGF promotes angiogenesis, enhancing the ability of tumor cells proliferation, invasio, and metastasis (Zhao et al., 2022). M2-like TAMs can directly affect IGF, initiating the insulin/IGF1R signaling pathway in the paracrine secretion pathway and enhancing resistance to gemcitabine in cancer patients (Vella et al., 2023).

### 4.6 Interactions and functional roles of TAMs within the TME

TAMs and cancer stem cells (CSCs) engage in intricate bidirectional crosstalk that critically promotes tumor progression and confers resistance to therapy. CSCs modulate the polarization of TAMs through the secretion of various cytokines and exosomes. For instance, exosomes derived from glioblastoma stem cells (GSCs) have been shown to activate the immunosuppressive STAT3 signaling pathway, facilitating the differentiation of monocytes into an M2-like TAM phenotype. This process reinforces an immunosuppressive tumor microenvironment and contributes to CSC-mediated drug tolerance (Panda et al., 2024). On the other hand, M2-like TAMs employ diverse mechanisms to sustain and enhance CSC stemness. Specifically, cytokines secreted by TAMs—such as IL-6 and IL-1β—activate the STAT3 pathway in CSCs, leading to the upregulation of key stemness-related genes (including NANOG, SOX2, and OCT4) that promote self-renewal and chemoresistance (Clemente-González and Carnero, 2022). IL-1β further amplifies this signal by inducing additional IL-6 production within the microenvironment, establishing a sustained positive feedback loop (Shi et al., 2025). Moreover, TAM-derived TGF-β engages in cross-talk with STAT3 and other signaling cascades through both Smad-dependent and Smad-independent mechanisms, synergistically enhancing stemness and driving epithelial–mesenchymal transition (EMT) (Tripathi et al., 2016). Beyond soluble factors, TAMs also support CSCs through metabolic and cell-contact-dependent pathways. The production of prostaglandin E2 (PGE2) via COX-2 expression activates Wnt/β-catenin and NF-κB signaling in CSCs, promoting proliferation and stemness maintenance (Morsli et al., 2025; Jiang et al., 2022). Additionally, cathepsin B released by TAMs remodels the extracellular matrix and activates latent growth factors, indirectly supporting CSC function (Larionova et al., 2019). Direct cellular interactions further reinforce this support: CD90-mediated binding between TAMs and CSCs stimulates stemness-related programs, while CD47–SIRPα engagement delivers a “do not eat me” signal that protects CSCs from phagocytic clearance (Larionova et al., 2019; Chu et al., 2024). TAMs also influence CSC plasticity through exosomal transfer of oncogenic miRNAs—such as miR-21 and miR-221/222—which repress tumor suppressors and negative regulators of stemness, thereby enhancing CSC survival and self-renewal (Cao et al., 2017). Furthermore, M2-like TAMs contribute to metabolic reprogramming within the tumor niche, modulating pathways such as arginine metabolism to shape a microenvironment that supports CSC metabolic adaptations and functional properties (Hasan et al., 2022). Targeting the dynamic interplay between TAMs and CSCs represents a promising therapeutic avenue. Combination strategies—such as CSF-1R inhibition together with CD47 blockade—simultaneously deplete TAMs and enhance phagocytic activity against CSCs (Skytthe et al., 2020; Bausart et al., 2022). STAT3 inhibitors administered alongside radiation or temozolomide chemotherapy can mitigate resistance mechanisms driven by stemness pathways (Wang Z. et al., 2021; Kohsaka et al., 2012). Nanocarrier-based targeted delivery systems also offer potential to improve drug specificity and reduce off-target toxicity. These multi-modal approaches aim to disrupt the supportive niche provided by TAMs while directly attenuating CSC stemness, thereby overcoming treatment resistance and reducing the risk of tumor recurrence and metastasis.

TAMs participate in extensive and intricate cross-talk with various cellular constituents of the TME, playing a central role in facilitating immune evasion and tumor progression. A particularly important bidirectional interaction exists between TAMs and CAFs. TAM-derived TGF-β1 activates Smad3 signaling to induce macrophage–myofibroblast transition (MMT), generating myCAFs that exhibit tumor-promoting functions (Chung et al., 2021; Stouten et al., 2023). In return, CAFs—especially inflammatory CAFs (iCAFs)—secrete factors including IL-6, CCL2, and CXCL12, which promote monocyte recruitment and their differentiation into M2-like polarized TAMs (Wang M. et al., 2025; Yang et al., 2024). This reciprocal activation is further reinforced through molecular axes such as COL1A1–CD44, contributing to sustained fibrotic and immunosuppressive niche formation. Consequently, excessive ECM deposition and remodeling occur, creating a physical barrier that restricts T cell infiltration and function.

TAMs also employ multiple mechanisms to suppress antitumor immunity. They express immune checkpoint ligands such as PD-L1 and secrete immunosuppressive cytokines including IL-10 and TGF-β, directly impairing the activity and expansion of CD8^+^ T cells (Liu et al., 2021). Additionally, TAMs modulate CAF activity and ECM organization, fostering an immune-excluded TME phenotype. By releasing chemokines like CCL22, they recruit regulatory T cells (Tregs), further amplifying local immunosuppression (Wang Y. et al., 2025). TAMs may also influence the formation and function of tertiary lymphoid structures (TLS), though their exact role—pro-inflammatory or anti-inflammatory—is context-dependent (Flores-Borja et al., 2016).

The complexity of cellular crosstalk within the TME often limits the efficacy of single-target therapies. Thus, current investigative efforts emphasize combination strategies targeting multiple components simultaneously. Examples include: biomimetic nanoparticle systems (e.g., SAB-PLGA@RTM) for co-delivery of agents that suppress both CAF activation and TAM polarization, while enhancing CD8^+^ T cell recruitment (Cheng et al., 2025; Zhou et al., 2025); combining CSF-1R inhibitors with immune checkpoint blockers to counteract TAM-mediated immunosuppression (Katoh, 2016); targeting signaling pathways such as TGF-β, JAK/STAT3, and CXCR4/CXCL12 to disrupt pro-tumorigenic communication networks (Liu et al., 2019); and emerging modalities including CAR-M therapies and efferocytosis-modulating nanotechnologies (Zhao et al., 2025; Zhou et al., 2021).

Future studies should leverage multi-omics approaches—single-cell RNA sequencing, spatial transcriptomics, and proteomics—coupled with computational modeling to decipher the heterogeneity and dynamics of TME interactions. There is also a need to develop condition-responsive drug delivery platforms that enable spatially and temporally controlled release of therapeutic agents within the TME. Identifying predictive biomarkers for patient stratification will be essential to advance personalized combination therapies aimed at reprogramming the TME and sustaining antitumor immunity.

### 4.7 Microenvironmental adaptation

Adaptive changes in the TME, such as CSF1R inhibitors, occur after treatment with targeting macrophages, suggesting that TAMs play an important role in regulating the tumor response to drugs (Zheng et al., 2018). Microenvironmental adaptations are reflected mainly in the adaptation of tumor cells to nutrient and oxygen deficiencies and in the effects of interactions between different cell types (CAFs and immune cells) (Nakahara et al., 2023). HIF1α promotes glycolysis (Warburg effect), allowing tumor cells to exist in an acidic environment (Nicolini and Ferrari, 2024). Study shows that acidic extracellular environment can trigger sterol regulatory element binding protein (SREBP), thereby contributing to tumor progression (Kondo et al., 2017). During the interaction of TAMs with CAFs and other immune cells, remodeling of the extracellular matrix can be promoted, and apoptosis escape can be facilitated (Shi et al., 2024). Understanding the adaptations of the TME, which plays an important role in tumorigenesis, progression, and metastasis, is crucial for the development of effective cancer treatment strategies. By targeting specific components of the microenvironment, novel therapeutic approaches can be developed.

### 4.8 Extracellular matrix remodeling

Under physiological conditions, remodeling of the extracellular matrix is an essential process for tissue development and repair. For example, during wound healing, remodeling of the ECM promotes the formation of new blood vessels and cell regeneration (Zhang W. et al., 2021). However, under pathological conditions, such as in tumors or fibrosis, aberrant remodeling of the ECM can lead to disruption of tissue structure and loss of function (Lin and Davis, 2023). TAMs are actively involved in extracellular matrix remodeling, cooperating with CAFs to promote the infiltration of tumor cells (Shi et al., 2024). M2-like TAMs activate fibroblasts to transform into myofibroblasts, promote the secretion of large amounts of collagen, and facilitate matrix deposition and ECM remodeling by regulating the balance of MMPs and their inhibitors (Simoes et al., 2020). During this process, the overproduction and cross-linking of collagen increase matrix stiffness (Zhang J. Y. et al., 2021). Increased matrix stiffness not only promotes the conversion of macrophages to the M2 phenotype but also affects the permeability and distribution of drugs, leading to difficulties in effectively reaching tumor cells and thus reducing the therapeutic efficacy of treatment (Xiong et al., 2024).

## 5 Potential applications of TAMs in preventing drug resistance

### 5.1 Reprogramming the phenotype of TAMs

#### 5.1.1 TLR agonists

TLR agonists act by inducing the production of a variety of cytokines, such as TNF and IL. These cytokines play important roles in regulating the function of immune cells, promoting inflammatory responses, and enhancing antiviral and antitumor immunity (Rolfo et al., 2023). The activation of TLRs is usually achieved through two main signaling pathways, the MYD88-dependent pathway and the non-MYD88-dependent pathway (Kircheis and Planz, 2023). Most TLRs bind to their ligands and, through MyD88, form a complex that further recruits IL-1R-associated kinase (IRAK). Through the interaction of MyD88 and IRAK, the TLR signaling pathway activates the NF-κB and MAPK pathways (Sun H. et al., 2022). TLR3 and TLR4 can also initiate another signaling pathway through TRIF; this pathway mainly activates IRF3, which promotes the production of type I interferon and enhances antiviral immune responses (Chen et al., 2021). TLR agonists can induce reprogramming of TAMs by activating the corresponding signaling pathways, thereby altering their phenotype and function. For example, R848 (a TLR7/TLR8 agonist) effectively promotes M1-like reprogramming of TAMs, increases the levels of antitumor components and reduces the secretion of substances that suppress immune response (Khan S. U. et al., 2023).

#### 5.1.2 STING agonists

The STING pathway consists of cGAS and STING. cGAS is activated upon recognition of intra- and extracellular DNA, producing cGAMP, which subsequently binds to STING and induces a series of downstream signaling pathways (Zhang et al., 2024). Studies have shown that the use of STING agonists can convert M2-like TAMs into M1-like macrophages (Chen et al., 2024). For example, DMXAA, a mouse STING agonist, not only inhibited the tumor cell-induced upregulation of genes related to protumor M2 polarization but also strongly stimulated the expression of genes associated with antitumor M1 traits (Wang et al., 2022). The sequence-gated transformable nanoprecursor MpRTNP was developed with SD-208 encapsulation to create MpRTNP@SD, aimed at sequential modulation of the TME and activation of the STING pathway (Li Y. et al., 2024). By converting M2-like TAMs to M1-like macrophages, STING agonists not only enhance antitumor immune responses but also improve the TME, providing a new direction for cancer therapy.

#### 5.1.3 CD40 monoclonal antibodies

**FIGURE 3:**
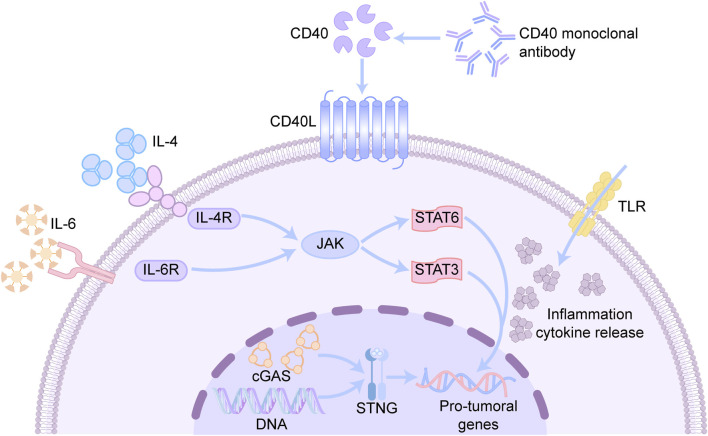
Mechanisms associated with reprogramming TAMs phenotypic drugs. CD40 is a cell surface receptor mainly expressed on antigen-presenting cells. CD40 monoclonal antibody can bind to CD40, activate downstream signaling pathways, and promote the activation and proliferation of immune cells. IL-4 is an anti-inflammatory cytokine, while IL-6 is a pro-inflammatory cytokine, which bind to IL-4R and IL-6R to activate the JAK-STAT signaling pathway respectively. STAT6 is a key transcription factor in the IL-4 signaling pathway, regulating gene expression and promotes Th2-type immune responses. STAT3 is a key transcription factor in the IL-6 signaling pathway, regulating inflammatory responses and cell proliferation. TLR recognizes pathogen-associated molecular patterns and initiates an innate immune response. CGAS senses DNA in the cytoplasm (e.g., pathogen DNA or DNA released by tumor cells) and catalyzes the production of cGAMP.

The binding of CD40 to its ligand CD40L activates antigen-presenting cells, and this binding promotes the maturation of antigen-presenting cells, cytokine production, and the activation of other immune-related functions (Mantovani et al., 2022). CD40 monoclonal antibodies can effectively inhibit tumor growth and strongly induce the expression of antitumor genes but decrease the levels of genes associated with protumor and tissue repair processes in TAMs (Beatty et al., 2011; Ho et al., 2014). CD40 signaling may trigger pro-inflammatory and antitumor macrophage polarization through reprogramming; the exact mechanism has not yet been elucidated but is closely related to mitochondrial metabolism (Figure 3). Inhibition of OXPHOS by rotenone or oligomycin was found to attenuate the induction of pro-inflammatory marker genes mediated by FGK45, an agonistic anti-CD40 monoclonal antibody (Liu et al., 2023). In addition, CD40 activation triggers alterations in fatty acid oxidation and glutamine metabolism, and alterations in these metabolic pathways promote ATP citrate lyase-dependent epigenetic reprogramming that supports the M1 phenotype (Liu et al., 2023; Lauterbach et al., 2019). CD40 monoclonal antibodies are often combined with other therapeutic agents, such as CSF-1R antibodies. These combination therapies can rapidly and profoundly reprogram TAMs before they are cleared, resulting in a synergistic effect (Enell Smith et al., 2021).

#### 5.1.4 STAT inhibitors

The STAT signaling pathway is a group of key signal transduction pathways involved in the regulation of the immune response, cell proliferation, and survival. STAT1 mediates M1 macrophage polarization through the IFNγ and TLR signaling pathways (Ma et al., 2018). The activation of STAT3 and STAT6 promotes macrophage polarization to the M2 type (Khan S. U. et al., 2023). Therefore, inhibition of the STAT3 and STAT6 pathways can achieve reprogramming of TAMs. Studies have shown that IL-6 expression plays a key role in glioma progression. IL-6 binds to its receptor and activates JAK2, which further phosphorylates STAT3, translocating it to the nucleus and regulating the expression of specific genes, a process that is an important mechanism by which glioma cells escape immune surveillance (Quan et al., 2024). Inhibition of STAT3 reduces the survival and proliferation of M2-like TAMs while promoting the activity of M1-like macrophages. IL-4 triggers JAK activation through IL-4R attached to cell membrane, which in turn activates IRS2 and PI3K, thereby recruiting and phosphorylating STAT6. Phosphorylated STAT6 directly binds to KLF-4 and PPAR-γ, and this binding promotes M2 macrophage polarization by initiating IRF4 (Wang S. et al., 2024; Kapoor et al., 2015). STAT inhibitors show great potential in reprogramming TAMs through a variety of mechanisms, such as blocking the JAK‒STAT signaling pathway, altering the cytokine secretion profile, regulating macrophage metabolism, and inducing apoptosis. STAT inhibitors can effectively transform the functional state of TAMs, thus providing new strategies for cancer therapy.

### 5.2 Metabolic reprogramming of TAMs

#### 5.2.1 Glucose metabolism

**FIGURE 4:**
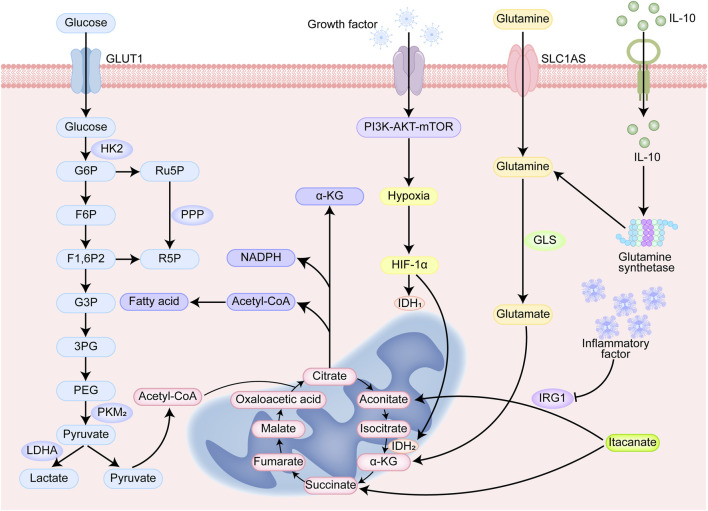
Cellular metabolism and immunoregulatory signaling pathways. Glucose enters the cell through GLUT1. Glucose is phosphorylated to G6P by HK2 and enters the glycolytic pathway. The glycolytic products F6P and G3P are further metabolized to generate pyruvate. Pyruvate can be converted to lactate by LDHA or enter the TCA cycle under aerobic conditions. G6P enters the PPP to generate Ru5P and R5P (ribulose-5-phosphate) and NADPH (reduced nicotinamide adenine dinucleotide phosphate). G6P can enter PPP to produce Ru5P and R5P, and at the same time produce NADPH for antioxidant and biosynthesis. α-KG is a key intermediate in the TCA cycle and participates in energy metabolism and biosynthesis. Glutamine is converted to glutamate by GLS, which is further metabolized to α-KG and enters the TCA cycle. Growth factor activates the PI3K-AKT-mTOR signaling pathway to promote cell growth, proliferation, and metabolism. HIF-1α regulates the expression of LDHA and PKM2, enhances lactate production and metabolic reprogramming. IL-10 is an anti-inflammatory cytokine that inhibits inflammatory responses.

The pathways associated with glucose metabolism include glycolysis, the pentose phosphate pathway (PPP), and the tricarboxylic acid (TCA) cycle (Figure 4). As part of the Warburg effect, tumor cells tend to produce lactate via the glycolytic pathway under aerobic conditions without participating in mitochondrial aerobic metabolism (Li and Tian, 2023).

M1-like TAMs are highly dependent on glycolysis in tumor cells. Tumor tissues experience a mostly hypoxic environment. Hypoxia-inducible factor (HIF)-1α is upregulated and promotes the expression of enzymes and factors related to glycolysis, which can be inhibited by the activation of the pyruvate kinase (PK) M2 tetramer, and the PKM2 dimer can upregulate the activity of HIF-1α (Jin et al., 2025). The Dectin-1/Akt/mTOR/HIF-1α pathway has been identified to be critical in the regulation of aerobic glycolysis, and the inhibition of mTOR-HIF-1α reduces glycolysis levels and eliminates training immunity (Hu et al., 2022).

Intermediates of aerobic glycolysis can be involved in the PPP, through which NO and ROS can be produced, both of which play key roles in the elimination of tumor cells by M1-like macrophages (Kennel and Greten, 2021). Moreover, RL5P, a metabolite of the PPP, enhances an ROS-mediated antimicrobial defense and selectively inhibits inflammatory responses (Das Gupta et al., 2023). Glycolysis and the PPP promote macrophage inflammatory responses through the mediation of NOX2 oxidase and IFNβ (Erlich et al., 2022). M2-like TAMs are dependent on mitochondrial metabolism and rely primarily on the TCA cycle for energy generation. The TCA cycle is interrupted in LPS-activated macrophages, mainly because inflammatory factors can upregulate aconitic acid decarboxylase 1 (IRG1), thereby promoting cis-aconitate (ITA) production (Choi et al., 2021; Michelucci et al., 2013). ITA is an inhibitor of succinate dehydrogenase (SDH), leading to the production of large amounts of succinate by macrophages during inflammatory activation (Ferreira et al., 2023). Accumulation of succinate stabilizes HIF-1α, leading to increased IL-1β production during inflammation (Tannahill et al., 2013). The abundant functional mitochondria and the intact TCA cycle in M2-like TAMs enable them to exhibit considerable adaptability, promoting their repolarization to the M1 state (Van den Bossche et al., 2016).

#### 5.2.2 Fatty acid metabolism

TAMs undergo extensive metabolic reprogramming of fatty acid metabolism to adapt to TME. In contrast to M1 macrophages, which predominantly rely on *de novo* fatty acid synthesis, TAMs adopt an M2-like phenotype characterized by dependence on fatty acid oxidation (FAO) for energy production (Oliveto et al., 2025). This shift is facilitated by the upregulation of the fatty acid transporter CD36, mediated in part by CD1d, leading to enhanced uptake of exogenous fatty acids and their storage as cytoplasmic lipid droplets (Zhou et al., 2024; Yang et al., 2022; Su et al., 2020). Excessive lipid accumulation suppresses mTORC2 signaling, contributing to an immunosuppressive phenotype by attenuating pro-inflammatory responses (Wu et al., 2019). The stored lipids are subsequently utilized through FAO, which becomes a major bioenergetic pathway in TAMs (Su et al., 2020). Key enzymes such as carnitine palmitoyltransferase 1A (CPT1A) are upregulated, promoting the entry of long-chain fatty acids into mitochondria for β-oxidation (Wang Y. et al., 2025). Metabolites derived from FAO, including acetyl-CoA, may further stabilize the M2-like transcriptional program through epigenetic mechanisms such as histone acetylation, enhancing the expression of typical M2 markers (Kuznetsova et al., 2024). Concurrently, activation of lipid-sensing pathways such as PPAR-γ reinforces M2 polarization, thereby promoting immunosuppression and tumor progression (Luo et al., 2017). Experimental inhibition of FAO has been shown to reverse the protumor phenotype of TAMs (Liu et al., 2021). Treatment with the CPT1A inhibitor etomoxir significantly restores effector T-cell function and restricts tumor growth (Tang et al., 2024). Similarly, intervention with ellagic acid-based nanocomposites effectively suppresses CPT1A expression, activates the AMPK–mTOR axis, increases secretion of the pro-inflammatory cytokine TNF-α, and reduces release of the anti-inflammatory cytokine IL-10, collectively shifting TAMs toward an immunostimulatory phenotype (Hussein-A et al., 2021; Sun et al., 2025). Furthermore, CD40 activation stimulates both FAO and glutamine metabolism in TAMs, modulating the NAD^+^/NADH ratio and inducing epigenetic reprogramming that favors pro-inflammatory gene expression and an antitumor phenotype. This metabolic rewiring is particularly evident in macrophages dependent on ATP-citrate lyase, underscoring the therapeutic potential of targeting metabolic pathways to reprogram the TME (Liu et al., 2023).

#### 5.2.3 Glutamine metabolism

Glutamine metabolism provides metabolites for several pathways that are activated primarily in the anti-inflammatory process. Stimulation of interleukin-10 (IL-10) plays a crucial role in enhancing the expression and activity of glutamine synthetase (GS). This relationship is important because IL-10 is known for its anti-inflammatory properties, and increased levels of GS help promote an anti-inflammatory environment in the body. In contrast, when the activity of glutamine synthetase in macrophages is inhibited, there is a notable shift from this anti-inflammatory state to a pro-inflammatory state. This transformation highlights the importance of GS in maintaining the balance between inflammatory and anti-inflammatory responses in immune cells, emphasizing its vital role in regulating inflammation (Palmieri et al., 2017). The metabolic process that produces α-ketoglutarate (α-KG) plays a key role in M2 polarization in macrophages. α-KG fuels the TCA cycle to increase FAO and OXPHOS in M2-like TAMs and drives epigenetic reprogramming of M2 genes in a histone methylation-dependent manner, and an increase in the α-KG/succinate ratio suppresses to suppress inflammatory gene expression (Liu et al., 2017; Oh et al., 2020). Research indicates that inhibiting glutamine catabolism exerts anti-tumor effects by directly targeting tumor cells and modulating immune cells within the tumor microenvironment. For instance, the glutamine metabolism inhibitor JHU083—a prodrug that releases its active compound DON upon activation within tumor tissues—demonstrated significant efficacy in both the ID8VEGF-high ovarian cancer ascites model and the iPAD endometrial cancer model (Li et al., 2025). Its anti-tumor mechanism is dual: it directly suppresses glutamine-dependent tumor cell proliferation while selectively reducing immunosuppressive M2-like TAMs and preserving pro-inflammatory M1-like macrophages (Li et al., 2025). This process is closely associated with the inhibition of HIF-1α and MYC signaling pathways. Additionally, the cytokine IL-16 indirectly suppresses glutamine catabolism by downregulating glutaminase (GLS) in CD4^+^ T cells, thereby promoting Th1 cell differentiation and IFN-γ secretion (Wen et al., 2025). IFN-γ further acts on TAMs, driving their reprogramming toward an M1 phenotype and upregulating the expression of chemokines CXCL9 and CXCL10, which enhances T cell recruitment and anti-tumor immune responses (Basu et al., 2021). Both direct and indirect regulation of glutamine metabolism can alter the phenotype and function of TAMs, thereby remodeling the tumor immune microenvironment and providing a theoretical foundation for combining metabolic intervention with immunotherapy.

### 5.3 Inhibition of TAM recruitment

#### 5.3.1 Drugs targeting the CCL2/CCR2 axis

Targeting the CCL2-CCR2 signaling axis provides a strategic approach to hinder the recruitment and activation of M2-like TAMs. By interfering with this pathway, monocytes can be retained within the bone marrow of M2-like TAMs in primary tumors and metastatic sites. Furthermore, inhibition of this axis is associated with an increase in the number of CD8^+^ T cells, which are crucial for effective anti-tumor immunity. Ultimately, these combined effects contribute to the suppression of tumor growth and invasion, highlighting the potential therapeutic benefits of manipulating the CCL2-CCR2 axis in cancer treatment (Urbantat et al., 2021a). MK-0812, a CCR2 inhibitor, can effectively block the binding of CCL2 to CCR2 and inhibit the activation of downstream signaling pathways to achieve good antitumor effects (Sugiyama et al., 2024). In addition, there are anti-CCL2 antibodies that can mediate the same effects (Lascano et al., 2023). In a rat model designed to study glioblastoma, researchers investigated the effects of the CCL2 inhibitor mNOX-E36. This inhibitor exhibits a dual impact: it not only impeded the recruitment of M2-like TAMs, but it also enhanced antivascular therapy against glioblastoma effectiveness. Despite these promising findings in the preclinical setting, it is important to note that the clinical efficacy of mNOX-E36 has not yet been confirmed through trials involving human subjects. Further research is necessary to determine its potential benefits in clinical applications (Cho et al., 2019). It is likely that in the complex TME, other chemokines or cytokines will compensate for the absence of CCL2 and CCR2 through negative feedback or other mechanisms and limit efficacy (Pienta et al., 2013). Therefore, the effect of blocking the CCL2‒CCR2 signaling axis needs to be further evaluated.

#### 5.3.2 Drugs targeting the CSF-1/CSF-1R axis

CSF-1 is a classical tumor-stimulating factor that is mainly secreted by fibroblasts, bone marrow stromal cells, and tumor cells. CSF-1R is a receptor synthesized by the c-Fms gene, and binding to CSF-1 results in dimerization of the receptor, which initiates signaling and ultimately promotes the proliferation, migration, and survival of M2-like TAMs (Denny and Flanagan, 2021). Preclinical studies showed that inhibiting CSF-1R considerably hindered glioma development and significantly boosted survival (Zomer et al., 2022; Akkari et al., 2020). The CSF1R inhibitor BLZ945 has proven highly effective in preclinical trials and is currently in phase I/II clinical trials in solid tumors, including glioblastoma (Watson et al., 2024). Although CSF-1R inhibitors have shown efficacy in targeting M2-like TAMs, the clinical results suggest that there are limitations to their antitumor effects; the TME initiates heterotypic paracrine secretion of the cytokine IL-34, which is produced by cancer cells, TAMs, and stromal cells and interacts with CD138 and PTP-ζ and promotes monocyte survival, proliferation, and differentiation, thereby promoting tumor growth and progression (Monteleone et al., 2023). The use of CSF-1R inhibitors in combination with other therapeutic strategies can remedy this deficiency.

#### 5.3.3 Intervention at the CXCL12/CXCR4 axis

CXCR12 is a natural ligand for CXCR4, and the CXCR4/CXCL12 pathway plays a crucial role in managing pathological mechanisms associated with human disease, including cancer, immune responses, and inflammatory responses. It induces the proliferation of various types of tumor cells through the activation of extracellular signal-regulated kinases and the AKT signaling pathway (Wang F. et al., 2024). The interaction between CXCR4 and CXCL12 leads to the accumulation of monocytes, as well as other cell types, in local tissues and affects the secretion of multiple cytokines (Zhang et al., 2023). Small molecule antagonists (e.g., plerixafor), peptides/peptide mimetics (e.g., BKT140), monoclonal antibodies (e.g., PF-06747143 and ulocuplumab), and microRNAs have shown promise in reducing the tumor load, inducing apoptosis, and rendering malignant cells resistant to conventional chemotherapy (Martino et al., 2024). Despite many advances in CXCR4 inhibitor research, several challenges remain. First, a major obstacle is how to effectively translate preclinical research results into clinical applications. Second, the side effects of CXCR4 inhibitors, such as cardiotoxicity and bioavailability issues, need to be further optimized and addressed. Researchers are exploring new combinations of inhibitors and modes of administration to improve therapeutic efficacy and safety.

### 5.4 Removal of M2-like TAMs

Inducing the apoptosis of M2-like TAMs is an effective strategy for removing them. Trabectedin (ET-743), an antitumor drug, has shown efficacy in removing TAMs; the main mechanism is the depletion of TAMs through the activation of exogenous apoptotic pathways by TRAIL receptors (Yang et al., 2020). CAR-T cell therapy is an innovative immunotherapy that works by genetically engineering patients’ T cells to enhance their ability to attack cancer cells. CAR-T cells can directly act on M2-like TAMs expressing folate receptor β (FRβ), leading to TME reprogramming, which results in the promotion of endogenous T-cell-mediated immunity and the control of tumor progression (Rodriguez-Garcia et al., 2021). In addition, drugs modified with M2pep can selectively kill M2-like TAMs and malignant cells but are less toxic to M1-like TAMs (Ngambenjawong et al., 2016). This selectivity makes M2pep an anticancer construct with a dual mechanism that kills malignant cells and removes tumor-supporting TAMs. There have been several clinical trials on M2-like TAMs treatment strategies to prevent drug resistance in glioma (Table 2). The removal of M2-like TAMs, while promising for tumor therapy, has potential drawbacks and challenges that should not be overlooked. Balancing the removal of tumor cells with the protection of normal cells is an issue that requires significant attention.

**TABLE 2:** Clinical studies on M2-like TAMs treatment strategies to prevent drug resistance in glioma.

Description	Intervention	Tumor type	gov identifier
Innate immune agonists			
TLR3 agonist	Poly (I:C)	Glioblastoma	NCT03392545
TLR7 agonist	Imiquimod	Diffuse midline glioma, H3 K27M-mutant recurrent high grade glioma	NCT06305910
	Resiquimod	Recurrent melanoma	NCT01748747
STING agonist	DMXAA	Advanced or recurrent solid tumors	NCT01285453
Immunostimulatory antibody			
CD40 monoclonal antibodies	Mitazalimab	Metastatic pancreatic cancer	NCT05650918
Intracellular signaling pathway inhibitors			
STAT3 inhibitors	Napabucasin	Colorectal cancer metastatic	NCT03647839
	Niclosamide	Acute myeloid leukemia (AML)	NCT05188170
Cell migration/chemotaxis inhibitors			
CCR2 antagonist	BMS-813160	Pancreatic cancer, CRC, NSCLC	NCT03184870
	PF-4136309	PDAC	NCT01413022
Macrophage-targeting inhibitors			
CSF-1 inhibitor	MCS-110	Melanoma	NCT03455764
	PD-0360324	Recurrent fallopian tube carcinoma Recurrent ovarian carcinoma Recurrent primary peritoneal carcinoma	NCT02948101
CSF-1R inhibitor	Pexidartinib	Tenosynovial giant cell tumor	NCT04703322
	Emactuzumab	Solid cancers	NCT02323191
	BLZ945	Advanced solid tumors	NCT02829723

### 5.5 Enhanced phagocytosis by macrophages

Signal regulatory protein α (SIRPα) is located on macrophages, while CD47 is present on a variety of healthy and tumor cells. The SIRPα‒CD47 axis protects tumor cells from the immune system by inhibiting phagocytosis by macrophages (Jin et al., 2024). Therefore, after blocking the SIRPα‒CD47 axis, macrophages will actively phagocytose cells lacking CD47-mediated protection, increase the phagocytic capacity of macrophages and inhibit tumor development. Owing to the ubiquitous expression of CD47, especially in hematopoietic cells, anti-SIRPα therapy targets only myeloid cells and has fewer side effects compared to anti-CD47 therapy (Liu et al., 2024b). Currently, selective SIRPα antagonists in clinical development include SIRPα-IgG and SIRPα-Fc fusion proteins and monoclonal antibodies targeting SIRPα; these antagonists enhance the phagocytosis of macrophages by specifically blocking the SIRPα/CD47 pathway and have shown potential therapeutic efficacy in different types of cancers (Son et al., 2022). In addition, bioengineered mesenchymal stem cells (MSC) activated by TGF β can secrete anti-tumor cytokines IL-12 and nCD47-SLAMF7 fusion proteins, which regulate T cell activity and macrophage phagocytosis (Li M. et al., 2024). SIGLECs are a class of glycoproteins that play important roles in the immune system, and they inhibit phagocytosis by macrophages by binding sialic acid (Xiao et al., 2021). Tumor cells transduce CD24, which can interact with SIGLEC10 and inhibit the phagocytosis of tumor cells (Barkal et al., 2019). Therefore, inhibiting the activity of SIGLEC molecules has been suggested as a potential strategy to increase phagocytosis in macrophages. Enhanced macrophage phagocytosis has shown promising clinical prospects and therapeutic potential as an effective tumor immunotherapy. Despite limited single-agent efficacy in solid tumors, combination therapies are expected to improve the therapeutic efficacy of these approaches.

## 6 Personalized treatment plan

Given the marked heterogeneity and complexity of gliomas, the development of personalized therapeutic strategies targeting TAMs necessitates a precision oncology approach founded on integrated multi-omics profiling. This process begins with a comprehensive assessment of individual genetic alterations—such as IDH mutation status and MGMT promoter methylation—coupled with detailed molecular profiling of oncogenic signaling pathways and tumor staging (Stumpo et al., 2022; Dorosti et al., 2025). Further refinement is achieved through high-resolution characterization of the TME using single-cell RNA sequencing, spatial transcriptomics, and multiplexed immunofluorescence, which collectively elucidate TAM subsets, their functional polarization, and spatial architecture (Le et al., 2025). Based on these insights, tailored therapeutic strategies can be devised: tumors exhibiting high TAM infiltration may benefit from “depleting” agents such as CSF-1R inhibitors, while immunologically “cold” tumors might be targeted with TAM-“reprogramming” approaches, including TLR or CD40 agonists, potentially combined with immune checkpoint blockade (Wu et al., 2024; Sato et al., 2025; Starska-Kowarska, 2023). Evaluating the efficacy and safety of these personalized regimens requires moving beyond conventional radiological criteria (e.g., RANO). Instead, it should incorporate advanced functional imaging (DWI, DSC-PWI) for early response assessment, longitudinal liquid biopsy analyses, and when feasible, repeat tissue sampling to verify TAM modulation. Concurrently, careful monitoring of immune-related adverse events (irAEs) and CNS-specific toxicities is essential. Together, these components form a cohesive framework that links molecular diagnostics, mechanism-based therapeutic intervention, and multidimensional response evaluation, thereby facilitating truly personalized glioma management.

## 7 Summary

Glioblastoma cells express myeloid gene program, suggesting TAMs play an important role in their drug resistance (Gangoso et al., 2021). Immunobiological therapeutic translation of TAMs involves the heterogeneity and adaptability of mononuclear phagocytosis in tumors. By analyzing the diversity and plasticity of TAMs, targeting the recruitment, activation, or function of TAMs can weaken their tumor-supporting effects and the response of tumor cells to drug treatment, providing new perspectives on strategies to selectively remove tumor-promoting and tumor-suppressing subpopulations. Although targeting TAMs is a promising therapeutic in preclinical experiments, few drugs have been successful in clinical trials. Given the complexity of the glioma microenvironment and the blood‒brain barrier, it is unrealistic to expect a single agent to have an adequate therapeutic effect. Therefore, these therapies can be used in combination with standard therapies or immunotherapies to inhibit tumor progression and improve patient prognosis. However, there are still challenges related to side effects, off-target effects, and drug resistance in the targeted treatment of TAMs. These challenges arise from multiple factors. Primarily, the pronounced heterogeneity of TAMs within the glioma microenvironment, coupled with their diverse cellular origins, poses significant obstacles to precise therapeutic targeting. This heterogeneity is exemplified by subpopulations such as Siglec-9^+^TAMs, which can be further stratified into Siglec-9^+^MARCO^+^and Siglec-9^+^SEPP1^+^subsets (Mei et al., 2023). Furthermore, broad-spectrum inhibitors, exemplified by CSF-1R blockers like PLX3397, exhibit limited efficacy against microglia-derived TAMs, contributing to suboptimal clinical outcomes (Peng et al., 2025). Compounding these issues, drug delivery is severely hampered by the blood-brain barrier, particularly for large-molecule therapeutics, and systemic administration incurs significant off-target effects; for instance, CSF-1R inhibitors can affect liver Kupffer cells, while targeting the CCL2-CCR2 axis may impair myocardial repair (Han et al., 2022; Guo S. et al., 2024). These off-target effects lead to dose-limiting toxicities that necessitate dose reduction, resulting in subtherapeutic concentrations at the tumor site. Additionally, the non-specific actions of epigenetic modulators further complicate therapeutic strategies. Overcoming these multifaceted challenges necessitates the development of subpopulation-selective targeting agents, optimization of strategies to enhance blood-brain barrier penetration, and the design of spatiotemporally controlled combination therapy regimens. Recent advancements in bionic nanocarrier systems have demonstrated significant potential in overcoming the limitations of the BBB and enhancing targeted therapy for gliomas. Among the promising strategies, a dual-stage neutrophil-mediated carrier system (termed “Trojanbot”) exhibited remarkable performance. The Trojanbot carrier synergistically integrates pH/ROS-responsive drug release with immune activation mechanisms, resulting in a 1.68-fold increase in CD8^+^ T cell infiltration within the tumor microenvironment and extending the median survival time of tumor-bearing mice to 38 days (Gao et al., 2025). Comparative studies further highlight the unique advantages of macrophage-based carrier systems. For instance, the macrophage-delivered MFe_3_O_4_-Cy5.5 platform significantly enhanced survival rates in animal models by integrating photothermal-guided surgery with therapy (Wang S. et al., 2021). Similarly, in a 3D glioma model, macrophage-mediated delivery of CPNs substantially improved the efficacy of photodynamic therapy (PDT) compared to free CPNs (CPNs-PDT system) (Ibarra et al., 2020). Furthermore, an innovative SPCFe/siP nano-therapeutic system leverages ultrasound activation to induce the ferroptosis pathway, representing a novel combinatorial therapeutic strategy (Liu Y. et al., 2025). Although many studies have used nanotechnology to improve the therapeutic efficacy of targeting, both passive and active targeting have limitations. Further exploration is needed to maximize the efficacy of targeted TAM therapy.
